# Polyethylenimine‐grafted mesoporous silica nanocarriers markedly enhance the bactericidal effect of curcumin against *Staphylococcus aureus* biofilm

**DOI:** 10.1002/jbm.b.35108

**Published:** 2022-06-23

**Authors:** Ayşenur Pamukçu, Nursu Erdoğan, Didem Şen Karaman

**Affiliations:** ^1^ Department of Biomedical Technologies, Graduate School of Natural and Applied Sciences Izmir Katip Çelebi University Izmir Turkey; ^2^ Department of Biomedical Engineering, Faculty of Engineering and Architecture Izmir Katip Çelebi University Izmir Turkey; ^3^ Pharmaceutical Sciences Laboratory, Faculty of Science and Engineering Åbo Akademi University Finland

**Keywords:** antibacterial, antibiofilm, curcumin, mesoporous silica, polyethyleneimine, *Staphylococcus aureus*

## Abstract

The recalcitrant nature of biofilms makes biofilm‐associated infections difficult to treat in modern medicine. Biofilms have a high vulnerability to antibiotics and a limited repertoire of antibiotics could act on matured biofilms. This issue has resulted in a gradual paradigm shift in drug discovery and therapy, with anti‐biofilm compounds being sought alongside new drug carriers. A potential solution to biofilm‐associated infections is to employ antibiofilm treatments, which can attack biofilms from many fronts. Nanocarriers are promising in this regard because they can be entrapped within biofilm matrix, target biofilm matrix, and provide local drug delivery to inhibit biofilm formation. In this study, curcumin as an herbal extract was loaded onto hyperbranched polyethylenimine‐grafted mesoporous silica nanoparticles (F‐MSN‐PEI/Cur) and antibiofilm investigations were performed. The F‐MSN‐PEI/Cur design has the potential to repurpose curcumin as an antibiofilm agent by increasing its solubility and lowering the required doses for the destruction of matured biofilms as well as suppressing biofilm development. Using imaging and spectroscopic techniques, we assessed the interaction of F‐MSN‐PEI/Cur with *Staphylococcus aureus* bacterial cells and determined the impact of F‐MSN‐PEI/Cur on eradicating matured biofilms and suppressing biofilm development. The F‐MSN‐PEI/Cur design is highly cytocompatible, as observed by the cytotoxicity screening investigations on L929 mouse fibroblast cell line. Our findings show that F‐MSN‐PEI/Cur design reduces the bacterial cell viability, inhibits biofilm formation, and induces biofilm eradication, which is attributed to F‐MSN‐PEI/Cur design having the potential to repurpose the antibiofilm activity of curcumin‐herbal extract.

## 1 INTRODUCTION

Infectious diseases caused by bacteria have been life threatening due to increased resistance to antibiotics and the intricacy of the infection. Disease progression and the response of the host to the infection reveal two different clinical conditions, acute versus chronic infection.^1^ Chronic infections are highly associated with aggregated/attached clusters of planktonic cells, termed the biofilm mode of growth, with a delayed healing process.^2^ A biofilm is a structured bacterial community embedded in a three‐dimensional matrix that attaches to a solid surface or aggregates into clusters. The self‐produced, so‐called extracellular polymeric substances (EPS) matrix is responsible for the protection and structural stability of bacteria cells which also results in tolerance to antimicrobial treatments and environmental stress. Antibiotic tolerance in biofilms may be due to (i) EPS components preventing antibiotic diffusion through biofilms, (ii) metabolic activity and growth rate gradients within the biofilm restricting antibiotic uptake by bacteria cells, and (iii) antibiotic degradation by enzymes secreted by biofilm bacteria.^3^ Therefore, biofilms persist with antibiotic therapy up to 1000 times more than required for planktonic bacteria treatments.^4^ The emergence of biofilm‐associated infections has increased the demand for novel approaches for delivering bactericidal drugs through biofilms. One intriguing strategy is to use nanoparticles (NPs) to deliver not only antibiotics but also natural bactericidal agents to handle the issue of biofilm‐associated infections, which account for more than 60% of chronic infections in people.^3^

With the support of gained knowledge from research in oncology‐related nanomedicine, NPs have become attractive for the treatment of infectious diseases. Among existing NP, mesoporous silica nanoparticles (MSN) can provide multifunctionality (i.e., inherently therapeutic, drug delivery, targeted delivery, precision in dosing) due to their modular design options.^5^ The recent advancements in NPs aided antibacterial treatments and already well‐established research on MSN‐aided oncology treatments^6^, ^7^ have driven our interest in achieving MSN bactericidal delivery system for the treatment of biofilm‐associated infections due to the unique conceptual similarities between biofilms and tumor microenvironments.^5^ In comparison to the currently used antibacterial NPs for bacterial growth inhibition, modular MSN‐based bactericidal carriers may offer distinct advantages, including entrapment and penetration within the biofilm matrix via attachment to the EPS outer surface followed by migration into the biofilm. Furthermore, silica chemistry can be exploited to provide surface modification on MSN surfaces once for the benefit of high cargo loading, penetration through the biofilm matrix and better interactions with bacterial cells. In this regard, the allocation of primary, secondary, and tertiary amines on MSN surfaces via polyethylenimine (PEI) surface grafting is a legitimate strategy to deliver permeabilizing effects and disintegrate the bacterial cell membranes.^8^, ^9^ As a result, MSN‐PEI design can function as antibacterial agent carriers while also improving therapeutic efficacy by avoiding antimicrobial agent recognition and deactivation in the biofilm matrix, providing targeted delivery to cells, and increasing local concentration and antibacterial activity of bactericidal agents against bacterial cells. MSN‐PEI drug carriers have the potential to improve the efficacy of bactericidal agents by repurposing existing medications and minimizing the development of drug resistance, primarily by lowering the drug concentration required for the treatment and clearance of biofilm infections.^8^

Curcumin, the primary component of turmeric, has been shown to possess a variety of pharmacological properties and to exert antibacterial activity when combined with other antibacterial agents.^9^, ^10^, ^11^, ^12^, ^13^ Although curcumin's bactericidal property is imposed in combination with other compounds, its use alone is hampered by its low water solubility, stability in physiological environment, and bioavailability.^14^ It is classified in Class IV according to Biopharmaceutical Classification System (BCS) and its usage results in low absorption, inadequate distribution, and rapid elimination from living systems.^15^, ^16^, ^17^ It was indicated once the curcumin is orally administered (up to 12 g/day) less than 1% of it culminated in human circulation system.^18^ Moreover, curcumin has been noted as having very low water solubility of only 0.6 μg/ml and is susceptible to degradation particularly under alkaline conditions.^19^ The use of curcumin is limited due to low water solubility under acidic or neutral conditions, high decomposition rate in alkaline media and photodegradation in organic solvent.^20^ More in detail, the degradation of curcumin in series of pH conditions ranging from 3 to 10 has been shown and the result revealed that decomposition was pH‐dependent and occurred faster at neutral‐basic conditions.^21^ In vitro studies showed that more than 90% of curcumin tends to degrade in 30 min under physiological pH conditions (0.1 M phosphate buffer solution, 37 °C, pH 7.2).^22^ Surfactants, polymer mixtures, and the formation of inclusion complexes encompass the most used and successful approaches employed in the literature.^23^ Obvious approach to improve the biopharmaceutical properties of curcumin is to overcome water solubility limitations of curcumin by employing nanocarriers.^24^ In light of our previous study wherein we have shown the loading of curcumin into MSN possessing different surface chemistries could improve the interaction of curcumin without degradation due to the presence of surface‐grown PEI on MSN by providing strong interaction between PEI and curcumin.^25^ As such, the use of polyethylenimine surface grafted MSN (MSN‐PEI) as a carrier for curcumin is anticipated to be a promising strategy for repurposing curcumin as an antibiofilm agent by improving the water solubility and without providing combinatory antibiotic treatments.

In this study, we interpreted the potency of hyperbranched polyethylenimine (PEI) modified MSN as the curcumin carrier for inhibiting biofilm formation and eradicating matured biofilms. The interaction of F‐MSN‐PEI/Cur and planktonic bacterial cells was evaluated to resolve the rationale behind the antibiofilm efficacy of curcumin‐loaded fluorescently labeled MSN‐PEI (F‐MSN‐PEI/Cur). Our findings show that nanocarriers design, PEI surface grafted MSN significantly increase the bactericidal effect of curcumin while also providing operability in eradicating matured *S. aureus* biofilms and inhibiting the formation of *S. aureus* biofilm.

## 2 MATERIALS AND METHODS

### 2.1 Materials

Tetraethyl orthosilicate (TEOS, reagent grade, 98%, Sigma‐Aldrich) and aminopropyl triethoxysilane (APTES, Sigma‐Aldrich) were used as silica sources in the synthesis of MSN. Absolute ethanol (99% purity, IsoLAB), cetyltrimethylammonium bromide (CTAB, Merck), sodium hydroxide (Merck) and ammonium nitrate (Carlo Erba) were used as received for MSN synthesis. Milli‐Q water (18.2 MΩ cm) was used throughout the study. Fluorescein isothiocyanate (FITC) from Sigma‐Aldrich was used for the fluorescent labeling of MSN. Curcumin was purchased from Sigma‐Aldrich. Tyriptic Soy Broth (Merck), PBS buffer saline (Sigma‐Aldrich), Dulbecco's Modified Eagle Medium (Lonza BioWhittaker) (with 25 mM HEPES buffer and 4.5 g/L glucose) with an addition of 10% heat‐inactivated fetal bovine serum (FBS) (Gibco, Thermo Scientific), 2 mM l‐glutamine (Sigma‐Aldrich) and 10 μl/ml of penicillin–streptomycin (Sigma‐Aldrich) was used in in vitro studies. The *S. aureus* strain DSM 20321 was provided by Dr. Tuba Müderris from the Department of Medical Microbiology, Izmir Katip Celebi University.

### 2.2 Preparation of curcumin‐loaded fluorescently labeled mesoporous silica nanoparticles

**SCHEME 1:**
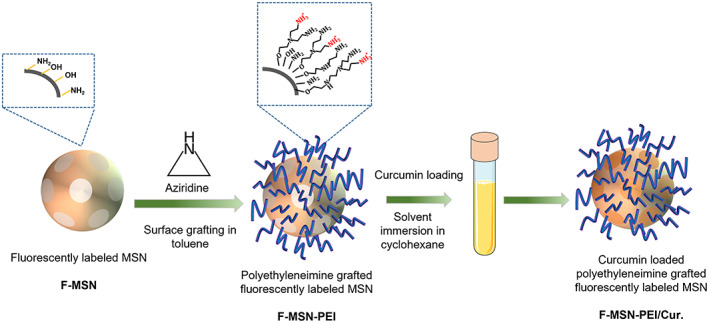
Schematic presentation for the reaction steps of F‐MSN‐PEI/Cur.

Fluorescein isothiocyanate (FITC)‐labeled MCM‐41‐type MSN synthesis was performed employing the synthesis protocol in our previous study.^26^ In brief, a basic aqueous solution was prepared with the addition of absolute ethanol (20% vol/vol) as a co‐solvent in the presence of the structure‐directing agents cetyltrimethylammonium bromide (CTAB), tetraethyl orthosilicate (TEOS), and aminopropyl ethoxy silane (APTES) as silica sources. The reaction was carried out at 33°C overnight. The molar composition of the synthesis solution was 1 TEOS: 0.01 APTES: 1.23 × 10^−2^ CTAB: 0.31 NaOH: 71.8 EtOH: 1063.8 H_2_O. Before reaction, APTES modification with FITC was performed by reacting FITC with APTES in absolute ethanol at a molar ratio of 1:3 and stirring for 2 h to provide successful incorporation within the silica framework. After the overnight reaction, the structure‐directing agent (CTAB) was removed by solvent extraction using an ethanolic NH_4_NO_3_ solution (20 g/L) and the obtained NPs, namely F‐MSN, were vacuum dried to be used for hyperbranched PEI surface modification. The surface modification of MSN with hyperbranched PEI by surface‐initiated polymerization was carried out according to the literature protocols.^27^ and the schematic presentation for the PEI surface grafting on F‐MSN and curcumin loading was presented in Scheme 1. To initiate PEI polymerization from the MSN surfaces, aziridine was used as a monomer with toluene as the solvent, in which the MSN substrate was suspended in the presence of catalytic amounts of acetic acid. The suspension was refluxed under atmospheric pressure overnight at RT, filtered, washed with toluene, and dried under vacuum at 313 K. Henceforth, the obtained fluorescently labeled NPs will be abbreviated as F‐MSN‐PEI. After obtaining F‐MSN‐PEI, dried samples were taken for curcumin loading. Curcumin was loaded into F‐MSN‐PEI by the solvent immersion method to obtain a final design of F‐MSN‐PEI/curcumin, named as F‐MSN‐PEI/Cur.^25^ In brief, sonication was used to disperse 20 mg of F‐MSN‐PEI in 10 ml of cyclohexane. Then, curcumin with an initial concentration of 50% wt/wt was added to the F‐MSN‐PEI suspension and then sonicated and allowed to vortex for 24 h at room temperature. The next day, the curcumin‐loaded F‐MSN‐PEI (F‐MSN‐PEI/Cur) was centrifuged and dried in an oven..

### 2.3 Physicochemical characterizations of F‐MSN‐PEI/Cur

The dried, extracted and PEI surface‐grafted MSN as the curcumin carrier has been subjected to physicochemical characterization to explore the features of the F‐MSN‐PEI/Cur and the carrier F‐MSN‐PEI design. The size distribution of resuspended dry F‐MSN‐PEI in HEPES buffer at pH 7.2 was investigated by subsequent dynamic lights scattering (DLS) measurements (ZetaSizer NanoZS 90, Malvern). The success of one‐step hyperbranching polymerization of a surface‐grown PEI was confirmed by ζ potential measurements (ZetaSizer NanoZS 90, Malvern) in HEPES buffer solution. The morphology of F‐MSN‐PEI was investigated by scanning electron microscope (SEM; Carl Zeiss 300VP). The F‐MSN‐PEI samples were dried under vacuum and then transferred onto a sample holder coated with gold (QUORUM Q150 RES).

Curcumin loading degree was determined by UV–VIS spectrophotometer. For this purpose, F‐MSN‐PEI/Cur samples were dispersed in ethanol at the concentration of 0.25 mg/ml by ultra‐sonication and kept in the sonication bath for 30 min, left for complete elution on rotating wheel overnight.^25^, ^28^ After eluting the adsorbed curcumin from F‐MSN‐PEI/Cur samples in ethanol, the NPs were centrifuged and supernatants were taken for spectrophotometric investigation. The absorbance value at the highest peak point of the 425 nm wavelength was recorded and the calculations were made by employing the curcumin standard curve in ethanol at various concentrations (1–50 μg/ml) with the same instrument and experimental parameters. Net surface charge and hydrodynamic size of F‐MSN‐PEI and F‐MSN‐PEI/Cur were determined, respectively, with ζ potential and DLS analyses using NanoZS 90 Zetasizer (Malvern). For measurements, the samples were dispersed in HEPES buffer (25 mM, pH 7.2) at a concentration of 0.25 mg/ml.

After size and morphology investigations, F‐MSN‐PEI/Cur powders and curcumin were taken for curcumin solubility investigations to affirm the benefit of F‐MSN‐PEI to improve the bioavailability of bactericidal curcumin. This particular investigation was employed at high curcumin content, which was foreseen as the needed dosing in investigating the bactericidal effect of curcumin for biofilm inhibition and eradication. The curcumin solubility investigations were performed by following the literature protocols. The solubility of curcumin was performed in buffer solution at pH 7.2 by adding 0.1% (wt/vol) Tween 80 to restrain errors due to poor solubility which is a frequently used strategy in literature studies while working with low soluble molecules.^29^, ^30^, ^31^, ^32^, ^33^ Tween 80 (Sigma‐Aldrich) was used as an surfactant and a non‐ionic emulsifier to prevent aggregation due to poor solubility of curcumin at physiological pH.^34^ In detail, F‐MSN‐PEI/Cur powders were suspended as 320, 640 μg/ml in aqueous release media (HEPES, pH 7.2, 25 mM containing 0.1% (wt/vol) Tween 80) possessing curcumin content of 57.6 and 115.2 μg/ml and was taken into Float‐A‐Lyzer G2 dialysis device (MWCO:3.5–5 kDa) to investigate the improved solubility of curcumin by F‐MSN‐PEI carrier. The counterpart investigations were performed also for curcumin powder, which has equivalent curcumin content in the carriers. Briefly, 320 and 640 μg/ml F‐MSN‐PEI/Cur samples were immersed in aqueous media containing with a 0.1% (wt/vol) Tween 80. The curcumin solubility from the F‐MSN‐PEI/Cur was investigated by employing a UV–visible spectrophotometer. After 24 h, F‐MSN‐PEI/Cur suspension from the Float‐A‐Lyzer G2 dialysis device was centrifuged for 10 min at 10,000 rpm and supernatants were measured by Synergy HTX Multimode Plate Reader for solubility examination. The solubility of curcumin was calculated with a standard curve comprising various curcumin concentrations in the release medium. The same investigations were performed also for free curcumin in immersed media where the dialysis bag was immersed for 24 h. The solubility was determined in three replicates and measured three times. The results were statistically analyzed with two‐way ANOVA, followed by Sidak's multiple comparison test using GraphPad Prism (version 8.4.2). In order to solidify F‐MSN‐PEI nanocarriers‐aided curcumin solubility enhancement whether it is due to the obtained differences in the solid state of the curcumin crystals on nanocarriers, differential scanning calorimetry (DSC) investigations were also performed for curcumin powder, F‐MSN‐PEI and F‐MSN‐PEI/Cur powders. All samples were scanned by a scanning rate of 5 K/min. An empty aluminum crucible was used as a reference sample and the temperature scale of the calorimeter. A quantity of 2–6 mg (total weight) was used for the DSC measurements.

### 2.4 In vitro cytotoxicity investigations of F‐MSN‐PEI/Cur

Cytocompatibility assessment of F‐MSN‐PEI/Cur was investigated in the L929 mouse fibroblast cell line. Fibroblast cells were cultured in a basal medium comprised of Dulbecco's Modified Eagle Medium (DMEM), 10% FBS, and 1% penicillin–streptomycin. Furthermore, the cells were seeded onto a standard cell culture plate at a concentration of 10^4^ cells/well and incubated overnight at 37°C in 5% CO_2_ environment to allow cell attachment. The following day, F‐MSN‐PEI/Cur was dispersed in filtered HEPES buffer and diluted with the basal medium at a concentration of 10, 25, 50, 100, and 200 μg/ml. Fibroblast cells were treated with diluted F‐MSN‐PEI/Cur and, subsequently incubated at 37°C with 5% CO_2_ for 24, 48, and 72 h. After incubation, treated cells were rinsed gently with sterile phosphate buffer saline (PBS) to remove NPs. Cell viability was determined by the 3‐[4,5‐dimethylthiazol‐2‐yl]‐2,5 diphenyl tetrazolium bromide (MTT) assay. Serum‐free DMEM containing 10% (vol/vol) MTT stock solution (5 mg/ml) was added to treated cells and then incubated at 37°C. After incubation, formazan crystals were dissolved using dimethyl sulfoxide (DMSO) and absorbance values were measured at 570 nm by Synergy HTX Multimode Plate Reader.

### 2.5 In vitro antibacterial and antibiofilm investigations of F‐MSN‐PEI/Cur

#### 2.5.1 Determination of bacterial growth inhibition by F‐MSN‐PEI/Cur

*Staphylococcus aureus* (DSM 20321) have been employed as a gram‐positive model organism as a resemble of bacterial strains causing infection.^35^, ^36^ To investigate the impact of F‐MSN‐PEI/Cur on the planktonic growth form of the bacterial cell, the growth profiles of F‐MSN‐PEI/Cur‐treated bacterial cells were investigated. For this purpose, the optical density values of the F‐MSN‐PEI/Cur treated bacteria during 18 h were compared to the untreated bacteria suspensions. In detail, *S. aureus* culture was grown overnight by inoculation of tryptic soy broth (TSB) from a single colony at 37°C and 150 rpm under aerobic conditions. The following day, the overnight grown cultures were diluted at 1:50 in TSB for 2 h to reach the exponential growth phase. Subsequently, the *S. aureus* culture concentration was adjusted to 10^8^ CFU/ml by optical density measurement at 600 nm (OD 600) using Synergy HTX Multimode Plate Reader. The bacteria suspension was then diluted in TSB to a final concentration of 2 × 10^5^ CFU/ml. A 100 μl of F‐MSN‐PEI/Cur (the final concentrations of 25, 50, 100, 200, 400, 800, and 1600 μg/ml) were pipetted into the wells of 96‐well microtiter plates. Subsequently, 100 μl of 2 × 10^5^ CFU/ml *S. aureus* suspension was added to each well. The plate was incubated at 37°C for 18 h and OD 600 measurement was carried out with 30 min intervals. Non‐treated bacteria were used as negative control while F‐MSN‐PEI/Cur suspensions in TSB without bacteria were used as the blank of the actual samples. By this way, we eliminated the optical density interference arising from components of F‐MSN‐PEI/Cur, that is, curcumin and FITC and also arisen due to the colloidal status of the particle suspensions. Three replicates were conducted for each group. Subsequently, the bacterial viability assessment was conducted for the same set of investigations to depict the bactericidal efficacy of F‐MSN‐PEI/Cur on the planktonic growth form of the bacterial cell. However, in this particular investigation, the bacterial cell suspensions were treated with an increasing concentration of F‐MSN‐PEI/Cur at 37°C for 5 h to investigate the bacterial cell viability. After incubation, resazurin solution (200 μM, resazurin sodium salt, Sigma‐Aldrich) was added to each well at a final concentration of 10% (vol/vol). The plate was then incubated at 37°C in a dark environment. Fluorescence measurement was carried out at excitation/emission wavelengths of 530/590 nm using CLARIOstar® multimode reader. Non‐treated bacteria suspensions were employed as negative controls, while F‐MSN‐PEI/Cur suspensions without bacteria were used as blank to eliminate F‐MSN‐PEI/Cur induced backgrounds.

#### 2.5.2 Determination of biofilm inhibition and eradication degree after F‐MSN‐PEI/Cur treatments

##### Inhibiting the biofilm formation by F‐MSN‐PEI/Cur treatments

The inhibition of biofilm formation by F‐MSN‐PEI/Cur and equivalent free curcumin content was performed by treating the bacteria cells with increasing concentrations of F‐MSN‐PEI/Cur suspensions and equivalent quantities of free curcumin solutions during the of biofilm formation. In detail, overnight culture of *S. aureus* was set to10^8^ CFU/ml by OD 600 measurement followed by dilution in TSB supplemented with 0.2% (vol/vol) glucose to obtain a concentration of 2 × 10^5^ CFU/ml. Afterwards, 100 μl of F‐MSN‐PEI/Cur (the final concentrations 400, 800, and 1600 μg/ml) and in parallel 100 μl of curcumin solutions (the final concentrations 72, 144, and 288 μg/ml as equivalent amount in F‐MSN‐PEI/Cur treatments) were pipetted into the wells of 96‐well microtiter plates. Then, 100 μl of 2 × 10^5^ CFU/ml *S. aureus* suspension was pipetted into the wells to obtain final *S. aureus* bacterial concentration 10^5^ CFU/ml. Suspensions in the wells were gently mixed by pipetting to provide a homogeneous dispersion of the F‐MSN‐PEI/Cur. For the negative control, 10^5^ CFU/ml *S. aureus* suspension was also incubated. The plates were incubated for 24 h at 37°C and 150 rpm. The quantification of the biofilm viability and biomass were performed after the treatments.

##### Eradicating the matured biofilm by F‐MSN‐PEI/Cur treatments

The eradication of mature *S. aureus* biofilms was also assessed with the same strategy. However, in this case, matured *S. aureus* biofilms were obtained as described above with only broth media. After mature biofilms were obtained on the following day, the medium in the wells was removed and the wells were washed with PBS three times to remove planktonic or loosely attached bacteria. Then, the ascending dose of F‐MSN‐PEI/Cur suspensions and free curcumin solutions were applied in parallel to assess biofilm eradication degree after F‐MSN‐PEI/Cur treatments, as well as the influence of PEI grafted MSN as a curcumin carrier. The mature biofilms were then treated with F‐MSN‐PEI/Cur suspensions of 400, 800, and 1600 μg/ml and in parallel equivalent free curcumin concentrations of 72, 144, and 288 μg/ml in HEPES buffer. Plates were incubated for 24 h at 37°C and 150 rpm to determine biofilm availability and total biomass of the treated biofilms. 24 h grown *S. aureus* biofilms started with a concentration of 1 × 10^5^ CFU/ml was used as a negative control. The quantification of biofilm viability and biomass were performed after the treatments.  

##### Biofilm inhibition and eradication degree analysis after F‐MSN‐PEI/Cur treatments

(i) Quantifying the biofilm viability and total biomass

To evaluate the degree of biofilm inhibition and eradication upon exposure to F‐MSN‐PEI/Cur suspensions and curcumin solutions, biofilm viability, and biomass were quantified using resazurin and safranin assays, respectively. To this end, after the treatments in Section 2.5.2 the supernatants in the wells were gently discarded, and biofilms were washed with PBS thrice to remove loosely attached bacteria. For biofilm viability analysis, 200 μl of 20 μM resazurin solution diluted in PBS was added to each well. The plate was incubated at room temperature and 150 rpm until a color change was observed in the negative control group. Fluorescence measurement was carried out at excitation/emission wavelength of 530/590 nm using CLARIOstar® multimode reader. For quantification of biomass, safranin dye was employed due to its known ability to bind to negative charges and therefore target many different molecules of bacteria and EPS.^37^, ^38^ After the treatments in Section 2.5.2 the supernatants in the wells were gently removed, and the biofilms were washed with PBS thrice. Thereafter, 200 μl of 0.1% (vol/vol) safranin solution was added to each well, and the plates were incubated at room temperature for 30 min. After incubation, safranin solutions were discarded, wells were washed with PBS twice to remove excess safranin, and the plates were air‐dried at room temperature. A 200 μl of 30% (vol/vol) glacial acetic acid was then pipetted into each well to dissolve safranin staining on biofilms. The biofilm mass was determined by measuring safranin absorbance at 525 nm using a Synergy HTX Multimode Plate Reader.

2 (ii) Estimating biofilm production ability of *S. aureus* during biofilm inhibition studies

The ability of the *S. aureus* cells for biofilm production after exposure to F‐MSN‐PEI/Cur suspensions was evaluated by employing the criteria described by Hassan et al. as demonstrated in Table 1.^39^ Biofilm production capacity of F‐MSN‐PEI/Cur treated bacterial cells was interpreted by using the OD_525_ values obtained from the safranin assay. For comparison, the optical density cutoff value (OD_c_) was calculated as mean (μ) OD of uninoculated TSB + 3 × *SD* (*σ*) of uninoculated TSB.

**TABLE 1:** Interpretation of biofilm production.

Average OD	Biofilm production
OD ≤ OD_c_	Non‐biofilm producer
OD_c_ < OD ≤ 2xOD_c_	Weak biofilm producer
2xOD_c_ < OD ≤ 4xOD_c_	Moderate biofilm producer
4xOD_c_ < OD	Strong biofilm producer

3 (iii) Investigating F‐MSN‐PEI/Cur entrapment within *S. aureus* biofilm

The degree of the F‐MSN‐PEI/Cur entrapment within the *S. aureus* biofilm was evaluated by the fluorescent intensity obtained due to FITC labeled F‐MSN‐PEI/Cur in order to ensure the diffusing ability of the F‐MSN‐PEI/Cur throughout to matured biofilm matrix.^40^ For this purpose, the matured *S. aureus* biofilms were prepared as described in Section 2.5.2. Then, F‐MSN‐PEI/Cur suspensions in HEPES buffer were added to wells containing *S. aureus* biofilms at three different concentrations (400, 800, and 1600 μg/ml). After 24 h., the suspensions on the biofilms containing non‐entrapped F‐MSN‐PEI/Cur in the wells were transferred to a 96‐well plate and the fluorescence emission was determined using the Synergy HTX Multimode Plate Reader at excitation/emission wavelengths of 485/528 nm. The experimental design used F‐MSN‐PEI/Cur suspensions in the absence of *S. aureus* biofilms at same treatment concentrations as those used in the presence of *S. aureus* biofilms as the starting point for fluorescent intensity measurements. To calculate the entrapped F‐MSN‐PEI/Cur concentration within the *S. aureus* biofilm, the difference between the initial F‐MSN‐PEI/Cur concentration and the non‐entrapped F‐MSN‐PEI/Cur concentration was calculated and used to calculate the F‐MSN‐PEI/Cur entrapment percentage. Afterwards,the concentration‐dependent entrapment efficiency of F‐MSN‐PEI/Cur was then calculated using the following equation as described by Devlin et al:^40^

$$F-M\mathrm{SN}-\frac{\mathrm{PEI}}{\mathrm{Cur}}\;\text{entrapment}\%=\frac{F_{c}-F_{e}}{F_{c}}\times 100$$

where *F*_c_ is the fluorescent intensity of the F‐MSN‐PEI/Cur suspensions, *F*_e_ is the fluorescent intensity of the F‐MSN‐PEI/Cur suspension entrapped within the *S. aureus* biofilms during 24 h. Therefore, the entrapped percentile of the added F‐MSN‐PEI/Cur suspensions within the *S. aureus* biofilms was evaluated.

4 (iv) Electron microscopy imaging of *S. aureus* biofilm after exposure to F‐MSN‐PEI/Cur treatment

The biofilms of *S. aureus* after exposure to the highest dose of F‐MSN‐PEI/Cur (1600 μg/ml) were investigated by scanning electron microscopy (SEM) for both inhibition and eradication studies to predict the architectural changes of the bacteria cells and biofilm matrix due to the F‐MSN‐PEI/Cur treatment. In detail, *S. aureus* biofilm was cultured in 24‐well plates containing sterile glass coupons with a starting concentration of 6 × 10^5^ CFU/ml with simultaneous addition of F‐MSN‐PEI/Cur for inhibition or addition of F‐MSN‐PEI/Cur to mature biofilm for eradication. After 24 h incubation, biofilms were washed with 1× PBS thrice to remove unattached bacteria and NPs. The *S. aureus* biofilms were then fixed in 2.5% (vol/vol) glutaraldehyde for 1 h., washed gently with PBS three times and air‐dried at room temperature, respectively. After fixation, the biofilm samples were gradually dehydrated with increasing concentrations of ethanol (25, 50, 70, 90, 100% (vol/vol)) for 20 min each. *S. aureus* biofilms were sputter coated with gold followed by visualization by Carl Zeiss 300 VP SEM instrument.

### 2.6 Data interpretation and statistical analysis

All experiments were carried out in triplicates and data are represented as mean ± *SD*. Statistical analyses were performed using GraphPad Prism software (version 8.4.2) with the level of significance (*α*) set to 0.05.

During all studies comprising biofilm inhibition, eradication and entrapment, biofilm, biofilm + curcumin and biofilm + F‐MSN‐PEI/Cur were also prepared as blanks for resazurin and safranin assays to eliminate biofilm, FITC, and curcumin‐induced interferences as the background. Nevertheless, some peaks for safranin were overlapped with curcumin peaks in absorbance spectra even after subtracting the blanks. This problem has been overcome by decomposition of the safranin and curcumin peaks using peak analyzer tool in OriginPro software (version 2019b) to process the area only under the safranin peaks for total biomass degree calculations.

The robustness of O.D. reading, safranin and resazurin assays were evaluated by calculating Z' factor, screening window coefficient, with the following equation:

$$Z^{\prime }=\frac{1-\left(3\times \sigma_{\max}+3\times \sigma_{\min}\right)}{\left|\mu_{\max}-\mu_{\min}\right|}$$

where *μ*_max_ and *μ*_min_ are the maximum and minimum mean of assay signals, and *σ*_max_ and *σ*_min_ are the *SD*s of the maximum and minimum assay signals. The test method is interpreted as appropriate if the Z' factor is between 0.5 and 1, acceptable in some cases if it is between 0 and 0.5, and not acceptable if the Z' factor value is <0.

## 3 RESULTS

### 3.1 Characterization of the F‐MSN‐PEI/Cur

**FIGURE 1:**
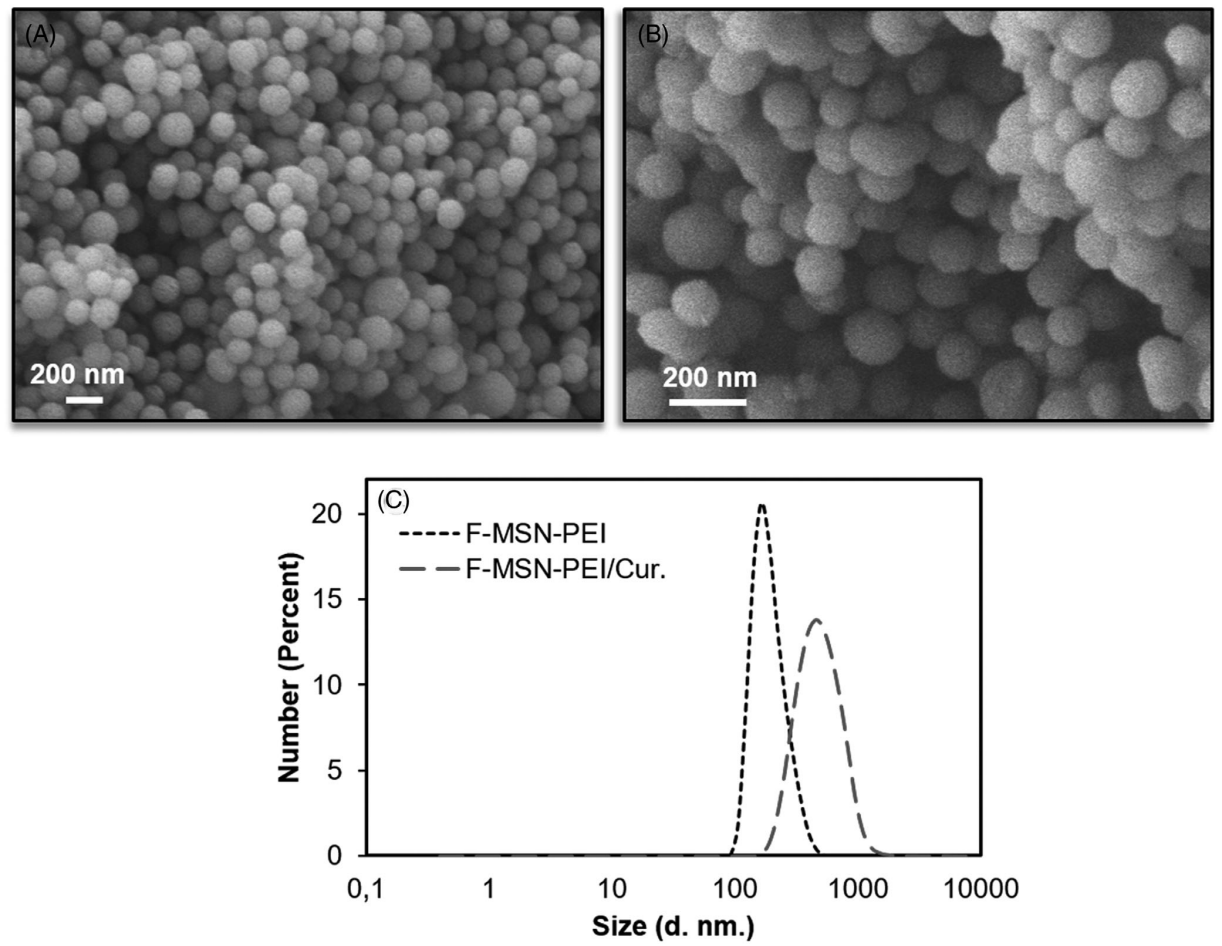
(A) SEM micrograph of F‐MSN‐PEI, (B) SEM micrograph of F‐MSN‐PEI/Cur, (C) Hydrodynamic size distribution of F‐MSN‐PEI and F‐MSN‐PEI/Cur.

The synthesis protocol yielded spherically shaped monodisperse F‐MSN‐PEI and F‐MSN‐PEI/Cur in the size range of 150–250 nm (Figure 1A,B). 250 μg/ml F‐MSN‐PEI and F‐MSN‐PEI/Cur NP suspensions were dispersed in HEPES buffer (pH 7.2, 25 mM) for hydrodynamic size and net surface charge (ζ‐potential) measurements, respectively (Figure 1C). The hydrodynamic size and ζ ‐potential data are presented in Table 2. The F‐MSN‐PEI samples were fully dispersible in HEPES buffer after all processing steps, including template removal, surface modification of F‐MSN with polyethylenimine (F‐MSN‐PEI) and further curcumin loading (F‐MSN‐PEI/Cur). The success of surface modification was ensured by the ζ‐potential measurements as presented in Table 2. The re‐dispersibility of F‐MSN‐PEI/Cur was investigated. The results obtained (Table 2) revealed that the loaded drug content does not cause an adverse effect on the re‐dispersibility of F‐MSN‐PEI even the hydrodynamic radius and the polydispersity index (PDI) values increased, in the acceptable range of PDI 0.1–0.4, which accounted for monodispersed size distribution. Furthermore, after curcumin loading, the ζ‐potential values could be produced with net positive values (+21 ± 4.8 mV).

**TABLE 2:** ζ potential, hydrodynamic radius and PDI results of F‐MSN and F‐MSN‐PEI/Cur. The results are expressed as mean ± *SD* (*n ≥* 3).

Characterization	F‐MSN‐PEI	F‐MSN‐PEI/Cur
ζ Potential (mV)	44.3 ± 6.5	21.2 ± 4.1
Hydrodynamic radius (nm)	236.1 ± 3.2	689.3 ± 106
Polydispersity index (PDI)	0.061 ± 0.02	0.316 ± 0.04

**FIGURE 2:**
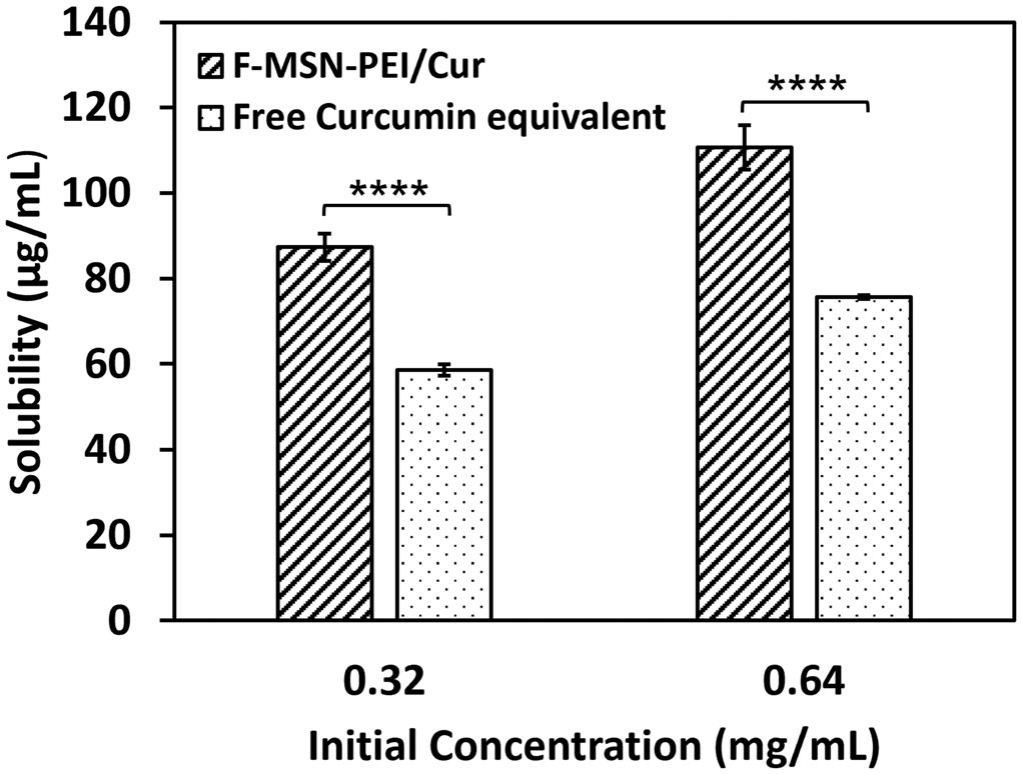
Solubility of curcumin in the F‐MSN‐PEI/Cur design in comparison to free equivalent curcumin content at different concentrations. The results are expressed as mean ± *SD* (*n ≥* 3). Statistical analysis was performed between F‐MSN‐PEI/Cur and equivalent curcumin at each concentration by applying two‐way ANOVA followed by Sidak's multiple comparison test. *p < .03, **p < .002, ***p < .0002, ****p < .0001

The degree of curcumin loading on F‐MSN‐PEI/Cur was determined to be 18% (wt/wt). The solubility of curcumin from the F‐MSN‐PEI carrier was evaluated to verify F‐MSN‐PEI is effective in enhancing curcumin solubility together with the commonly employed strategy of immersion into surfactant (i.e., non‐ionic Tween 80) added media.^23^ To understand the effect of F‐MSN‐PEI on curcumin solubility, solubilized curcumin with the aid of F‐MSN‐PEI nanocarriers at different concentrations of 320 and 640 μg/ml was investigated, and the free curcumin solutions were subjected to same spectrophotometric analysis. The solubility investigations were performed for F‐MSN‐PEI/Cur which contains 57.6 and 115.2 μg/ml curcumin and the counterpart free curcumin solutions at the concentration of 57.6 and 115.2 μg/ml. In this particular investigation, we have employed higher concentrations than the solubility limits, which was foreseen as the needed dosing in investigating the bactericidal effect of curcumin in the circumstance of biofilm inhibition and eradications. The observations showed that free curcumin solubility could be improved up to 58.6 ± 1.3 μg/ml and further 75.6 ± 0.4 μg/ml in 0.1% (wt/vol) Tween 80 (non‐ionic surfactant) aqueous solution once the starting concentration of curcumin increased.^41^, ^42^ In the literature investigations, researchers have claimed the solubility of curcumin as 0.6 μg/ml in water^19^ whereas in our investigations once the 0.1% (wt/vol) Tween 80 was added to HEPES buffer solution (25 mM, pH 7.2) the curcumin was solubilized 100‐fold higher than the literature finding. Our observations shown in Figure 2 indicated that when curcumin loaded onto F‐MSN‐PEI and immersed in the 0.1% (wt/vol) Tween 80 containing HEPES buffer the solubility of curcumin improved up to 110.6 ± 5.2 μg/ml which is almost 183‐fold higher 0.6 μg/ml where the solubility was investigated in water.^19^ This result could be ascribed as 0.1% (wt/vol) Tween 80 could aid the solubilization of curcumin however the incorporation of curcumin into F‐MSN‐PEI is more effective for improving the curcumin solubility compared to only 0.1% (wt/vol) Tween80 addition into immersion medium.

Furthermore, in order to solidify F‐MSN‐PEI nanocarriers‐aided curcumin solubility enhancement regardless of the presence of the solubility enhancer (Tween 80, non‐ionic surfactant presence) in the immersion media, DSC investigations were performed for the F‐MSN‐PEI/Cur and curcumin powders (Figure S2). Shifts of exothermic and endothermic peaks are usually associated with interactions between drug and carrier.^43^ However, in our investigations we have not observed a distinct shift in the melting point of curcumin, which could be indicated as the drug molecules could have been adsorbed via weak interactions by diffusing into pores and as aggregated in the PEI polymeric network of F‐MSN‐PEI carrier which may still lead the accommodation of both amorphous or disordered crystalline state. In Figure S2, a broader peak was observed for F‐MSN‐PEI/Cur which could be accepted as the indicative of a disturbance in the crystalline habit of the curcumin once in the F‐MSN‐PEI/Cur formulation which can be ascribed to a physical interaction between the excipients during processing. Almost 5‐fold ΔH reduction was also observed as given in Figure S2 for F‐MSN‐PEI/Cur compared to curcumin powder. This could be attributed to the disorganization of curcumin crystals after loading into F‐MSN‐PEI nanocarriers.^44^ The slightly lower T_m_ could be ascribed for this, while the ΔT_1/2_ remains unchanged.

### 3.2 In vitro cytotoxicity of F‐MSN‐PEI/Cur

**FIGURE 3:**
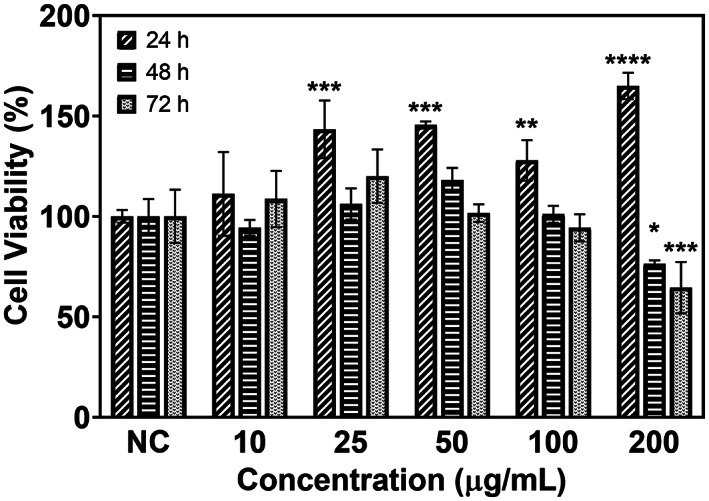
In vitro cytocompatibility of F‐MSN‐PEI/Cur with L929 cells. Error bars represent SD (*n ≥* 3). The results were statistically analyzed with the GraphPad Prism software (V 8.4.2) using two‐way ANOVA followed by the Dunnett multiple comparisons test. The degree of significance was *p < .03, **p < .002, ***p < .0002, ****p < .0001.

The cytocompatibility of F‐MSN‐PEI/Cur was evaluated and F‐MSN‐PEI/Cur was found to be cytocompatible at concentrations up to 100 μg/ml (Figure 3) over 24, 48, and 72 h incubation periods. When the dosing of F‐MSN‐PEI/Cur is increased higher than the 10 μg/ml actively proliferating cells were observed as shown in Figure 3 for 24 h treatments. However, the Figure 3 results reveal when the incubation time is increased the proliferative effect is not dominating as shorter term incubating.

### 3.3 In vitro antibacterial and antibiofilm investigations of F‐MSN‐PEI/Cur

#### 3.3.1 Bacterial growth inhibition and bactericidal effect of F‐MSN‐PEI/Cur

**FIGURE 4:**
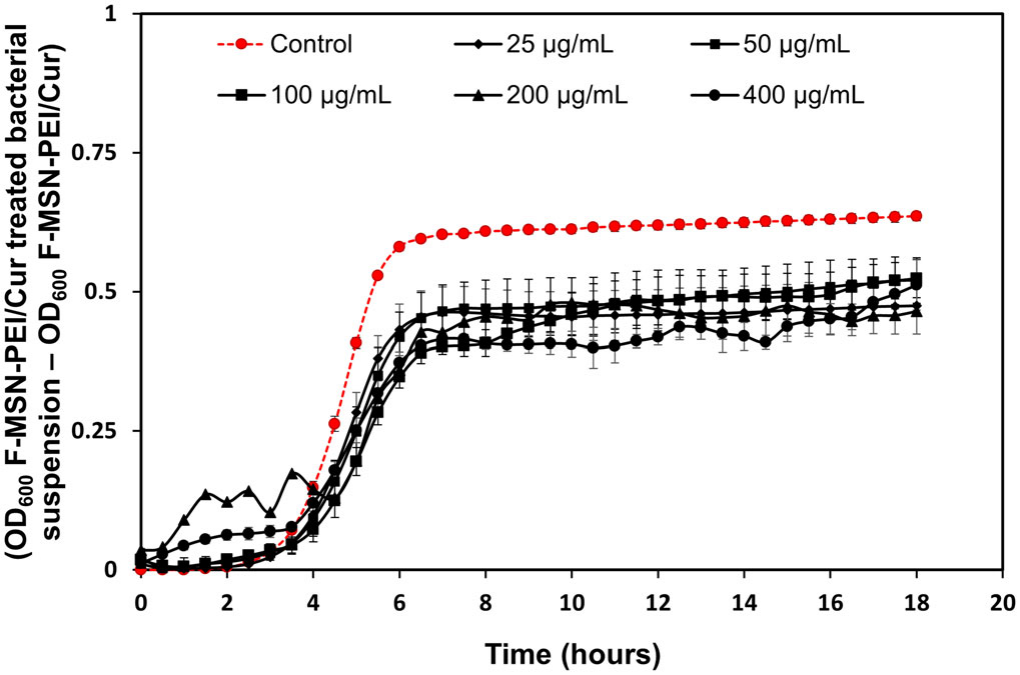
Growth profile of *S. aureus* cells upon exposure to ascending concentrations of F‐MSN‐PEI/Cur suspensions during 18 h to investigate the changes in growth profile in the presence of F‐MSN‐PEI/Cur. Error bars represent *SD* (*n ≥* 3).

The antibacterial activity of F‐MSN‐PEI/Cur were verified by incubating *S. aureus* suspensions with F‐MSN‐PEI/Cur followed by optical measurement (OD 600) of bacteria suspension. The optical density values of the F‐MSN‐PEI/Cur treated bacteria cell suspensions and untreated bacteria suspensions are presented in Figure 4. *S. aureus* bacterial cell growth profiles tends to change due to the F‐MSN‐PEI/Cur treatment. In order to clarify impact of the optical density changes due to the colloidal interference of the F‐MSN‐PEI/Cur we have followed the optical density of F‐MSN‐PEI/Cur suspensions in TSB (Figure S1). Obtained results showed drastic fluctuations and changes in optical density values when F‐MSN‐PEI suspensions higher than 400 μg/ml. The observed trend in Figure S1 could be due to the flocculation/sedimentation of the F‐MSN‐PEI/Cur content in the growth medium which may lead to the optical density fluctuations. In order to emphasize the elimination of NPs originated optical density disturbance the y‐axis of Figure 4 has been presented by subtracting the optical density values of F‐MSN‐PEI/Cur suspensions as the background from F‐MSN‐PEI/Cur treated bacterial suspensions. The lowest optical density values were obtained by 400 μg/ml treatment of the bacteria suspension (Figure 4) and the trend changes in the exponential growth phase were observed with the high concentration treatments (100, 200, and 400 μg/ml) compared to 25 and 50 μg/ml F‐MSN‐PEI treatments.

**FIGURE 5:**
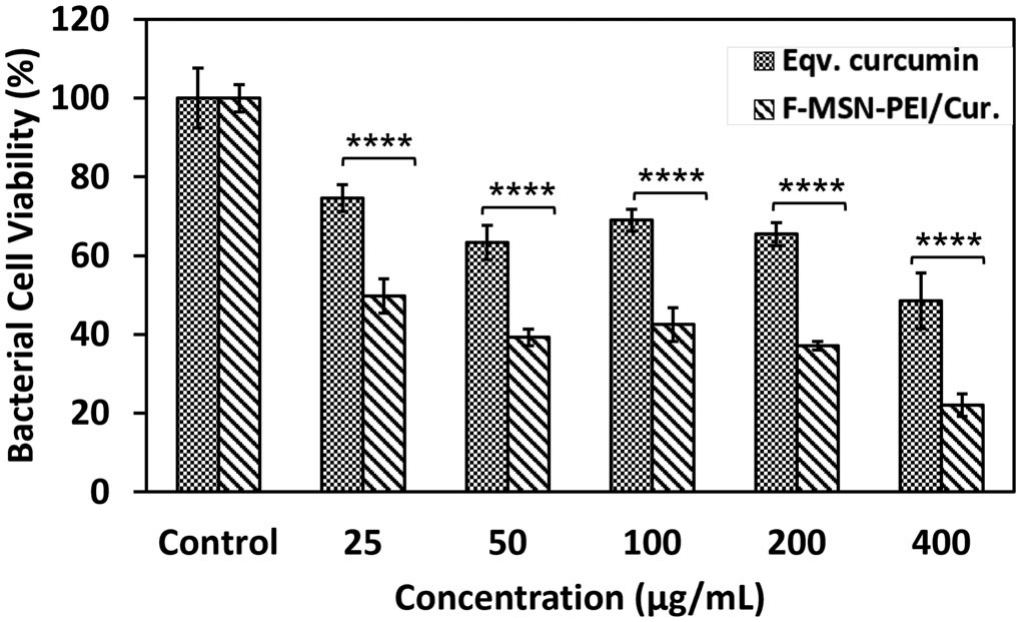
Bacterial cell viability investigation after 3 h treatment of bacterial suspensions of *S. aureus* with ascending F‐MSN‐PEI/Cur formulation. Statistical analysis was performed between F‐MSN‐PEI/Cur and equivalent curcumin at each concentration by applying two‐way ANOVA followed by Sidak's multiple comparison test. *p < .03, **p < .002, ***p < .0002, ****p < .0001

F‐MSN‐PEI/Cur and equivalent free curcumin amount loaded into F‐MSN‐PEI/Cur were then studied for its short‐term bactericidal action. The metabolic activity of the bacteria was studied using a resazurin assay after incubation of bacterial suspensions with increasing F‐MSN‐PEI/Cur and free curcumin dosing for 3 h (the beginning of the exponential growth phase of control bacteria). As the dose of F‐MSN‐PEI/Cur was increased, the metabolic activity of bacterial cells decreased, which is often related with reduced bacterial cell survival as shown in Figure 5. F‐MSN‐PEI/Cur dosing at 25 μg/ml inhibited bacterial cell viability by almost 50%, while higher dosings of 200 and 400 μg/ml reduced cell viability down to 38.03 ± 1.11% and 21.34 ± 2.87%, respectively. The treatment with curcumin, on the other hand, did not result in the same degree of cell viability reduction as seen in the Figure 5. However, the substantial interference by F‐MSN‐PEI/Cur in testing media was observed for the fluorescence reading of resazurin in these short‐term investigations. Therefore, we did not execute the investigations with the higher concentrations of 800 and 1600 μg/ml in this particular investigation.

#### 3.3.2 Biofilm inhibiting and eradicating efficiency of F‐MSN‐PEI/Cur

**FIGURE 6:**
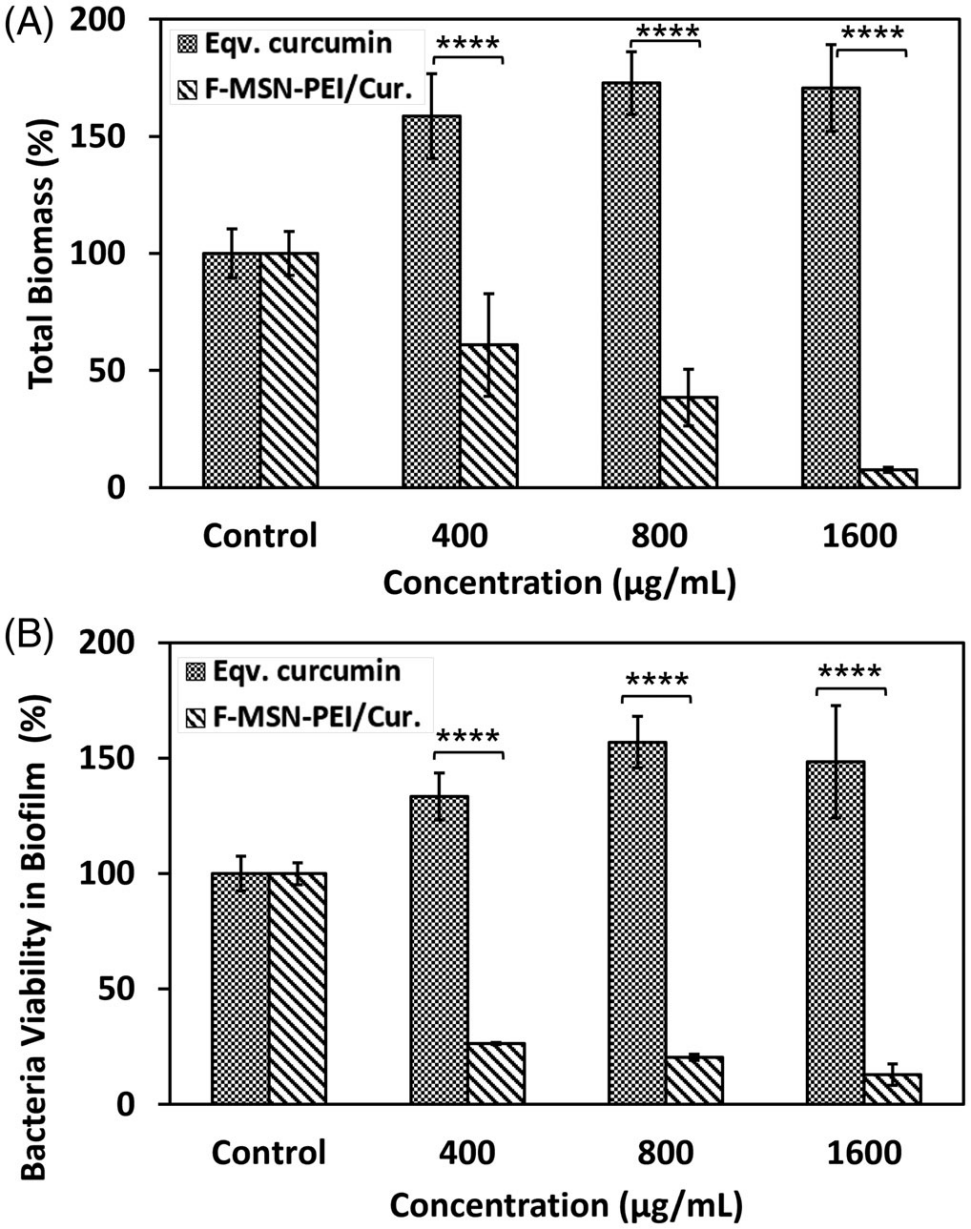
Investigation of the biofilm inhibiting potency of F‐MSN‐PEI/Cur and equivalent curcumin treatment by (A) safranin assay for total biomass and (B) resazurin assay for bacterial cell viability in biofilm. Statistical analysis was performed between treatment groups by applying two‐way ANOVA followed by Sidak's multiple comparison test, ns: 0.1234, *p < .03, **p < .002, ***p < .0002, ****p < .0001.

The impact of F‐MSN‐PEI/Cur and free curcumin treatments on inhibiting the *S. aureus* biofilm formation was evaluated. After 24 h treatments, the biofilms were quantified by analyzing total biomass change and the bacterial cell metabolic activity in biofilm matrix, compared to the untreated groups. The experiments were carried out with the 400–800 to 1600 μg/ml of F‐MSN‐PEI/Cur dosing and free curcumin equivalent to those in F‐MSN‐PEI/Cur designs. Figure 6 shows that almost 60% of the total biomass could be reduced when the bacteria cells were treated with 400 μg/ml of F‐MSN‐PEI/Cur dosing, whereas the highest dosing of 1600 μg/ml could reduce 90% of the total biomass compared to the negative control group. However, when we treated biofilm‐forming bacteria with curcumin, the total biomass and cell viability increment in biofilm matrix was observed in Figure 6 as a comparison to the effect of the F‐MSN‐PEI/Cur design. The obtained results indicated that 72, 144, and 288 μg/ml curcumin dosing without nanocarriers yielded almost 1.5‐fold more biomass compared to negative control groups. The observations indicated statistically significant differences between free curcumin and F‐MSN‐PEI/Cur groups.

**FIGURE 7:**
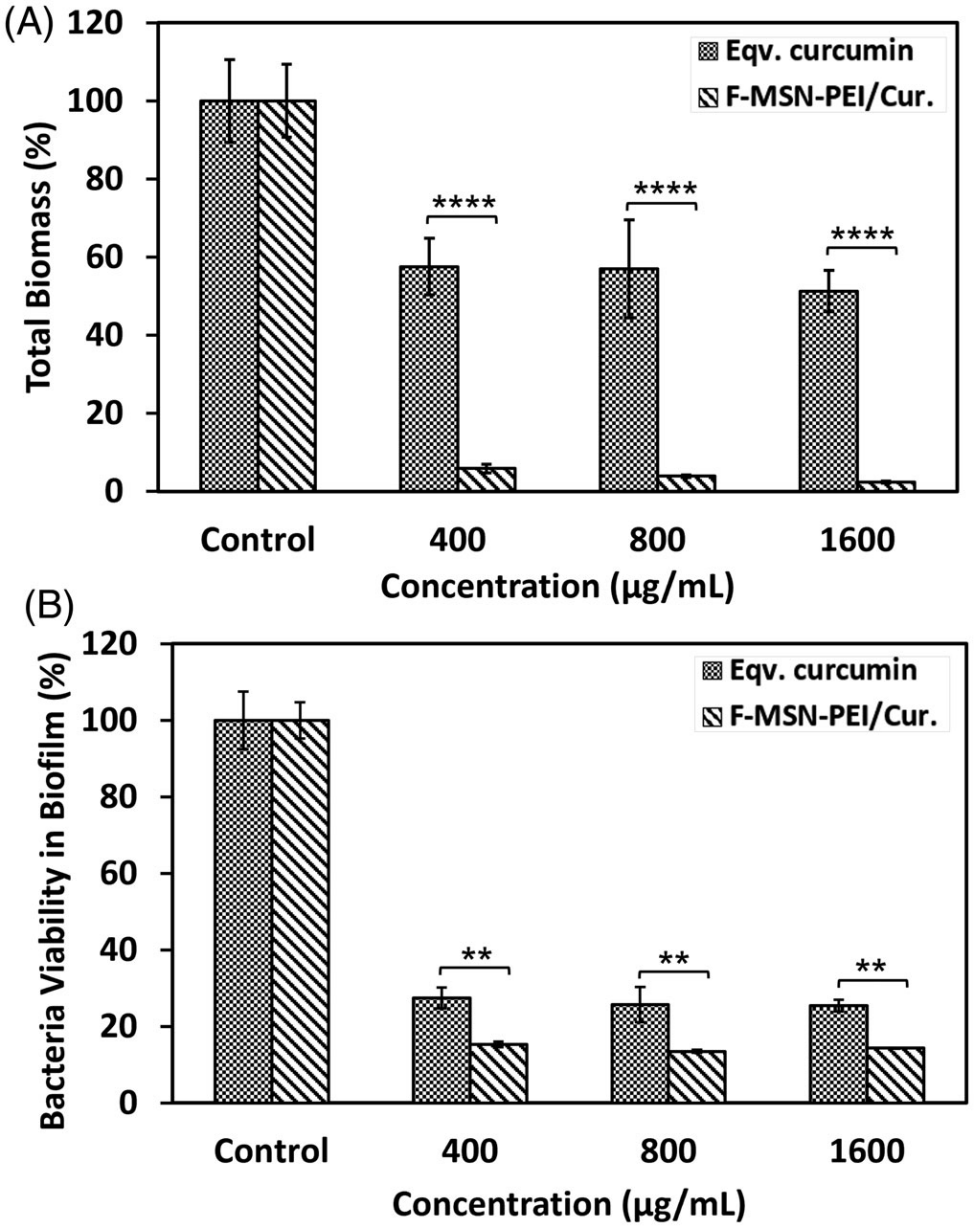
Investigation of biofilm eradication after F‐MSN‐PEI/Cur and equivalent curcumin treatment by (A) safranin assay for total biomass, (B) resazurin assay for bacteria cell viability. Statistical analysis was performed between treatment groups by applying two‐way ANOVA followed by Sidak's multiple comparison test, ns: 0.1234, *p < .03, **p < .002, ***p < .0002, ****p < .0001.

The efficacy of ascending concentrations of F‐MSN‐PEI/Cur and the free curcumin dosing on matured *S. aureus* biofilms are presented in Figure 7. The total biomass in Figure 7A showed that F‐MSN‐PEI/Cur treatments reduce the biomass significantly compared to free curcumin groups. The biofilm eradication potency of F‐MSN‐PEI/Cur is higher than free curcumin. However, equivalent dose of free curcumin treatment in eradication investigation worked more effectively compared to biofilm inhibition investigations. The reduction in the metabolic activity of the bacterial cells in the biofilm matrix was presented in Figure 7B. The obtained results showed statistically significant differences between free curcumin and F‐MSN‐PEI/Cur treated biofilm viability regardless of the increased dosing.

The Z′ factor, which predict the robustness of an assay, was calculated for the optical density reading assays used for bacterial growth profiles, safranin assay for biomass determination and resazurin assay for cell viability to affirm the separation between the distributions of positive and negative controls. The calculations resulted in the Z' factor between 0.1 and 0.6 for safranin assays, which is indicated as a moderate assay. However, the Z factor was calculated as 0.94 for optical density investigations and 0.84 for the resazurin assay, indicating the robustness and reproducibility of the assays.^45^

The biofilm forming capacity of the treated bacterial cells with ascending dosing of F‐MSN‐PEI/Cur were found that the treatment conditions completely eliminate the biofilm forming ability of *S. aureus* biofilms as presented in Table 3.^39^

**TABLE 3:** Interpretation of biofilm formation ability of F‐MSN‐PEI/Cur treated bacterial cells.

Treatments	OD_C_	OD	Interpretation
0 μg/ml	0.14	0.30	Moderate biofilm producer
400 μg/ml	0.74	0.17	Non‐biofilm producer
800 μg/ml	0.43	0.11	Non‐biofilm producer
1600 μg/ml	0.07	0.002	Non‐biofilm producer

**FIGURE 8:**
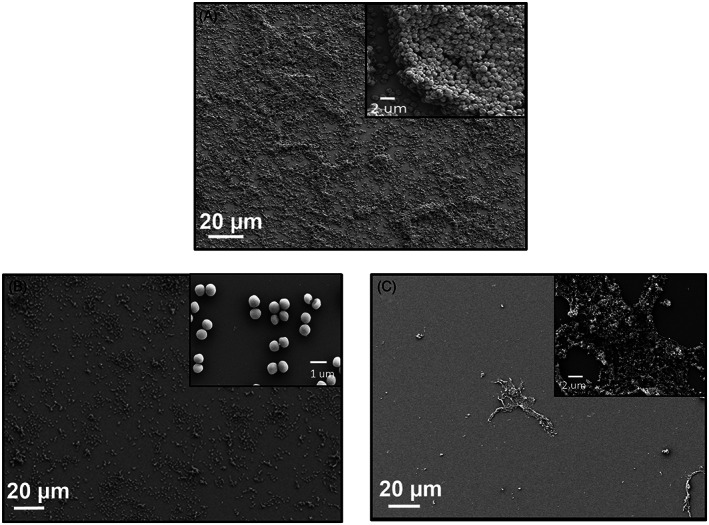
SEM micrograph of (A) untreated *S. aureus* biofilm, (B) F‐MSN‐PEI/Cur treated *S. aureus* bacteria during biofilm formation (C) F‐MSN‐PEI/Cur treated matured *S. aureus* biofilm.

SEM images of *S. aureus* biofilms after exposure to the highest dose of F‐MSN‐PEI/Cur (1600 μg/ml) for both inhibition and eradication investigations and negative control group were presented in Figure 8 to predict the architectural changes of the biofilm matrix after treatments. Reduction in the biomass for both cases was obtained compared to untreated condition. In addition, the debris of the biofilm matrix could be also visualized in Figure 8C whereas proper bacterium structures without any destruction could be visualized in Figure 8B.

**FIGURE 9:**
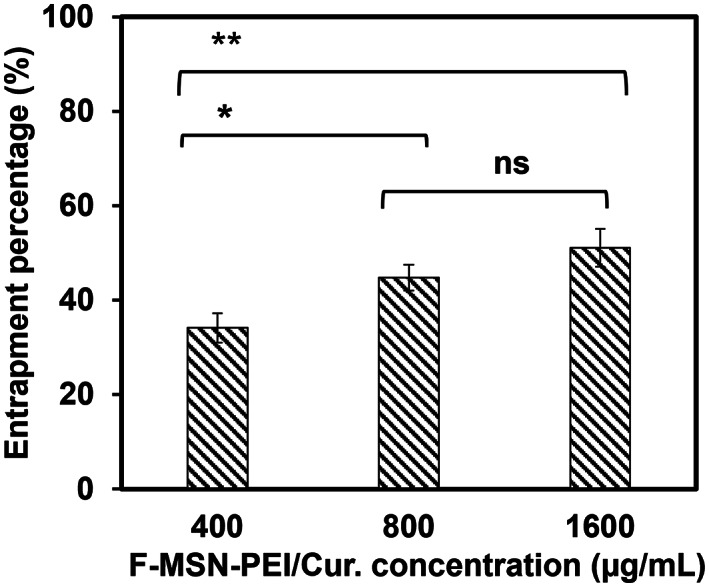
Entrapment efficiency of the F‐MSN‐PEI/Cur formulation into *S. aureus* biofilm. The results for entrapment efficiency were statistically analyzed in Graphpad Prism software (V 8.4.2) by ordinary one‐way ANOVA followed by Tukey's multiple comparisons test. The degree of significance for both graphs were ns:0.1234, *p < .03, **p < .002, ***p < .0002, ****p < .0001.

The F‐MSN‐PEI/Cur entrapment efficiency into the *S. aureus* biofilm was investigated to rationalize the correlation between the entrapment of F‐MSN‐PEI/Cur within the biofilm matrix and the biofilm eradication. After incubation of the matured biofilms with F‐MSN‐PEI/Cur suspensions, the fluorescence intensity of the supernatant containing F‐MSN‐PEI/Cur that have not yet entrapped into the biofilms and the control wells comprising the same concentration of F‐MSN‐PEI/Cur suspensions as starting concentration were measured. The obtained results are presented in Figure 9 demonstrating the entrapment efficiency as 34.11 ± 3.11%, 44.78 ± 2.76%, and 51.12 ± 3.99% for 400, 800, and 1600 μg/ml respectively. A significant increment in F‐MSN‐PEI/Cur entrapment could be observed when the dosing increased from 400 to 800 and 1600 μg/ml, however, the saturation of biofilm matrix with the F‐MSN‐PEI/Cur after the 800 μg/ml dosing was observed which could be related with the stacking of the F‐MSN‐PEI/Cur carrier more in the mid layer and upper layers of the biofilm matrix and not dose depended penetration through the biofilm matrix.

## 4 DISCUSSION

*S. aureus* is emerging as a problematic opportunistic pathogen that causes osteomyelitis, endocarditis, indwelling device infections, and wound infections.^46^ Due to the limited repertoire of antibiotics available today, there is a high demand for developing a new solution to overcome the failures of antibiotic treatments in *S. aureus* originated infections. The unique properties of NPs play an important role in inducing the antibacterial activity of antibiotics. Among the existing NPs, MSN provides a superior effect for cellular interactions. The use of MSN has been reported to be an effective antibiotic delivery system for antimicrobial agents. In the present study, the PEI surface grafted MSN as the carrier of herbal extract, curcumin, and the inhibition of planktonic and biofilm forms of *S. aureus* by F‐MSN‐PEI/Cur have been presented. F‐MSN‐PEI/Cur treatments both for the prevention of biofilm and eradication of matured biofilms was observed and affirmed by the reduction in biomass and cell viability in the biofilm matrix, which ultimately means the destruction of the biofilm‐forming capability of the cells and the biofilm microenvironments. These results could be derived from the increased local bioavailability of curcumin by the entrapment of the F‐MSN‐PEI/Cur within the biofilm matrix which is due to the penetration capability of F‐MSN‐PEI carrier through the matured biofilm matrix. The main mechanism of action in inhibiting *S. aureus* growth by curcumin was shown to be perturbation of bacterial membrane integrity.^13^ In literature studies, it was demonstrated that damaged bacterial membrane is correlated to impaired cell metabolism and cell death.^47^ Cell membrane damage in *S. aureus* after exposure to curcumin was also corroborated with electron microscopy images revealing distorted cell shape and cell debris. In addition, it was shown that curcumin induces damage in bacterial DNA as well by causing DNA fragmentation which is an indicative of apoptotic process.^48^ Even though the modes of curcumin‐mediated *S. aureus* cell killing were mainly investigated in planktonic form of bacteria in literature studies, the same mechanisms are possibly responsible for the anti‐biofilm action of curcumin. Increasing the bioavailability of curcumin by loading the curcumin on F‐MSN‐PEI carrier is therefore of great concern to obtain the desired anti‐biofilm effects since higher doses of the active ingredients is required against biofilms compared to planktonic bacteria.

In addition, the F‐MSN‐PEI carrier could aid in improving the biocompatibility of curcumin as a bactericidal agent. It was revealed that curcumin show distinct toxicity that is concentration dependent.^49^, ^50^ The incorporation of curcumin into F‐MSN‐PEI carriers did not cause any cytotoxicity and pungency, which could be due to both the combinatory effect of silica nanocarriers and improved solubility in aqueous physiological environments. Our results showed that curcumin loaded F‐MSN‐PEI supports fibroblast cell viability significantly during 24 h incubation. In the literature, different forms of silica have effect on cell behavior and the obtained results indicated that positively charged silica particles are taken up rapidly by cells and showed intracellular silicic acid release which resulted in positive effect on cell proliferation and migration.^51^, ^52^ Moreover, curcumin is known as highly effective for wound healing process. For instance, Dai et al. reported that human fibroblast HS‐27 cells incubated with cell culture media mixed extract from curcumin incorporated nanofibrous mats led to more proliferation than without curcumin containing mats and provide increased fibroblast migration hence, a higher wound closure rate inclined in vitro. In parallel, Kang et al. indicated that curcumin promoted the fibroblast proliferation until 12.5 μM/L.^50^ Similarly, in vivo wound healing results demonstrated that curcumin containing mats cause faster wound closure at 7 and 15 days^53^ and another in vivo study proved that curcumin showed thicker granulation tissue and greater re‐epithelization at 7 and 12 days of post operation.^54^ Additionally, the outer surface modification component of F‐MSN‐PEI design, PEI is well known for its cytotoxicity. Despite the toxicity consideration PEI functionalization is utilized commonly especially for gene delivery due to capability to employ reactive groups, increase stability, tune surface charge. It was indicated that PEI toxicity could be attributed to be molecular weight dependent.^55^ However, Desai and coworkers demonstrated that PEI adsorbed silica NPs in different forms showed less toxicity than PEI alone even at high concentrations. Also, toxicity did not correspond to the particles concentrations but PEI/SiO_2_ ratio. The study proved that incorporation of silica and PEI could aid to suppress the toxicity of PEI.^56^ All in all, the in vitro cytotoxicity investigations did not lead reduction in cell viability especially for 24 h incubation which is also matching with the treatment period in the bactericidal investigations.

F‐MSN‐PEI drug carrier could aid in improving the bactericidal effect of curcumin. Due to the spatial confinement of drug molecules within the F‐MSN‐PEI mesoporous network, drugs with poor solubility can be transformed into their amorphous/disordered crystalline counterparts with higher solubility.^57^ Also, it was shown that amine‐grafted mesoporous silica employed a prolonged and adjustable release profile of curcumin.^58^ In addition, the surface chemistry of the NPs has been shown to play a prominent role in altering the interaction between NPs and the biofilm matrix. This fact eventually determines the penetration ability, depth, and distribution of the NPs through the biofilm matrix.^59^ The complex nature of the biofilm matrix consists of different macromolecules with varying charges and hydrophobicity. Recently, researchers have shown that the penetration and localization of NPs through the biofilm matrix are highly dominated by the net surface charge of silica nanoparticles (SiNPs).^60^ Among the investigated surface properties of SiNPs, positively charged SiNPs resulted in the highest degree of entrapment, and hence the accumulation, by the biofilm matrix. Another consideration for NPs entrapment through biofilms is to determine which component (protein, eDNA, or polysaccharides) comprises the majority of the biofilm EPS matrix which has a direct effect on the affinity of the NPs. Studies investigating the relationship between the type of surface functionalization and different biofilm samples show that the entrapment degree of MSN within biofilm matrix can be tuned by the altered surface chemistry on mesoporous silica matrix.^61^ In our study, we showed that the polyionic nature of PEI on MSN carriers together with the loading of curcumin from the herbal extract (design‐MSN‐PEI/Cur) could provide a superior effect for inhibiting biofilm formation compared to free curcumin. This should be related to the polyionic nature of the PEI that could destruct the integrity of the bacterial cell membrane before bacterial cells reach the biofilm formation stages involving attachment of cells to a surface followed by the assembly of the cells to form micro colonies, and the differentiation of biofilm into a mature structure.^62^ In the literature, researchers have revealed that hydrophobic surfaces are more susceptible to bacterial cell adhesion than hydrophilic surfaces. Hence, the hydrophilic nature of F‐MSN‐PEI could inhibit the bacterial cell adhesion.^63^ In addition, the porous architecture of the silica matrix has been revealed to prevent the bacterial cell aggregations that take place as the initial step of biofilm formation, as also claimed in the literature studies.^64^ On the other hand, free curcumin treatment of bacterial cells during biofilm formation steps has induced the biomass formation compared to control groups, which could be due to the secretion of different components and could cause relapsing of the biofilm formation in the long term. When the short‐term treatment of *S. aureus* cells is taken into account, the viability of bacterial cells could already be reduced by 20% with 400 μg/ml F‐MSN‐PEI/Cur treatment compared to the negative control group, which could be depicted as that changes in the metabolic activity of bacterial cells could inhibit the biofilm formation potency of treated bacterial cells.

As presented in Table 3, F‐MSN‐PEI/Cur could play a role in inhibiting the biofilm‐forming capacity of the bacterial cells by switching them to non‐biofilm‐forming bacterial cells. However, there was only an acceptable level of statistical difference in the reduction of biofilm viability in the matured biofilms in Figure 7B, whereas the statistical differences were significantly higher in Figure 6B when the F‐MSN‐PEI/Cur and equivalent free curcumin treatments were compared. This could be related to the slow degradation of curcumin during the incubation time and/or stacking depth of F‐MSN‐PEI through *S. aureus* biofilm matrix. In our previous laboratory investigations as a part of another study, confocal imaging was performed for the F‐MSN‐PEI treated biofilms, and the plot of fluorescent intensity of F‐MSN‐PEI (count) versus depth of *S. aureus* biofilm (μm) after 24 h was plotted. The obtained analysis results of fluorescence count for 4, 8, and 14 μm depths of the biofilms revealed that F‐MSN‐PEI could penetrate through the deepest level of the biofilm matrix. However, the highest intensity, which could be depicted as the highest accumulation amount of F‐MSN‐PEI, could be achieved between 3 and 6 μm depths of the biofilm matrix. The accumulation of F‐MSN‐PEI/Cur in the middle layer could be due to the hydrophilic component that could overcome the hydrophobicity of the *S. aureus* cell membrane.^65^ This could be the reason for the higher accumulation of the F‐MSN‐PEI with the hydrophilic nature in the encapsulating layer of bacterial cells and could destroy the integrity of the EPS matrix, which leads mainly to a decrease in total biomass compared to the untreated preformed biofilms. Taken together, we showed that the antimicrobial and antibiofilm activities of curcumin can be enhanced by loading curcumin onto F‐MSN‐PEI nanocarriers. The F‐MSN‐PEI matrix as a drug carrier provides an opportunity for revolutionizing the bactericidal activity of curcumin, the common source of turmeric, for eradication of mature biofilms and prevention of biofilm formation without any combinatory antibiotic treatment.

## 5 CONCLUSION

The successful development of curcumin loaded PEI grafted mesoporous silica NPs nanocomposite, denoted F‐MSN‐PEI/Cur proves both antibacterial and antibiofilm activity. Short‐term treatments of *S. aureus* cells in planktonic form with F‐MSN‐PEI/Cur suspensions could provide a significant reduction in the cell viability even at low dosing (25 μg/ml) compared to free curcumin treatments, and the highest concentration of 400 μg/ml treatments could reduce the cell viability down to 20% compared to untreated control. These facts inhibit biofilm formation by reducing the viability of bacterial cells and total biomass in the biofilm matrix. Furthermore, increasing F‐MSN‐PEI/Cur dosing could prevent biofilm formation and eradicate matured biofilms compared to free curcumin treatments. Therefore, it is evident that PEI grafted mesoporous silica nanocarriers have great potency for repurposing of herbal extracts (i.e., curcumin) as anti‐biofilm agents against biofilms without any concomitant antibiotic treatment.

## Supporting information


**Figure S1** The optical density of F‐MSN‐PEI/Cur suspensions during 18 h incubation without bacterial cells.
**Figure S2**. DSC analysis of curcumin, F‐MSN‐PEI, F‐MSN‐PEI/Cur samples.

## Data Availability

Data available on request from the authors. The data that support the findings of this study are available from the corresponding author upon reasonable request.
