# Supplementary material for: Polyethylenimine‐grafted mesoporous silica nanocarriers markedly enhance the bactericidal effect of curcumin against Staphylococcus aureus biofilm
Source: J Biomed Mater Res B Appl Biomater. 2022 Jun 23;110(11):2506–20. doi: 10.1002/jbm.b.35108 (PMC9541607; doi:10.1002/jbm.b.35108)
Supplement: Supplementary file 1 — Figure S1 The optical density of F‐MSN‐PEI/Cur suspensions during 18 h incubation without bacterial cells. Figure S2. DSC analysis of curcumin, F‐MSN‐PEI, F‐MSN‐PEI/Cur samples. [file JBM-110-2506-s001.docx]

**Supplementary material for**

**Polyethylene imine-grafted mesoporous silica nanocarriers markedly enhance the bactericidal effect of curcumin against *S.aureous* biofilm**

Ayşenur PAMUKÇU,^a^ Nursu ERDOĞAN ^a^ and Didem ŞEN KARAMAN* ^b,c^

^a^ Department of Biomedical Technologies, Graduate School of Natural and Applied Sciences, Izmir Katip Çelebi University, Izmir, 35620,

^b^Department of Biomedical Engineering, Faculty of Engineering and Architecture, Izmir Katip Çelebi University, Izmir, Turkey

^c^ Pharmaceutical Sciences Laboratory, Faculty of Science and Engineering, Åbo Akademi University, Finland

*corresponding authors [didem.sen.karaman@ikcu.edu.tr](mailto:didem.sen.karaman@ikcu.edu.tr)


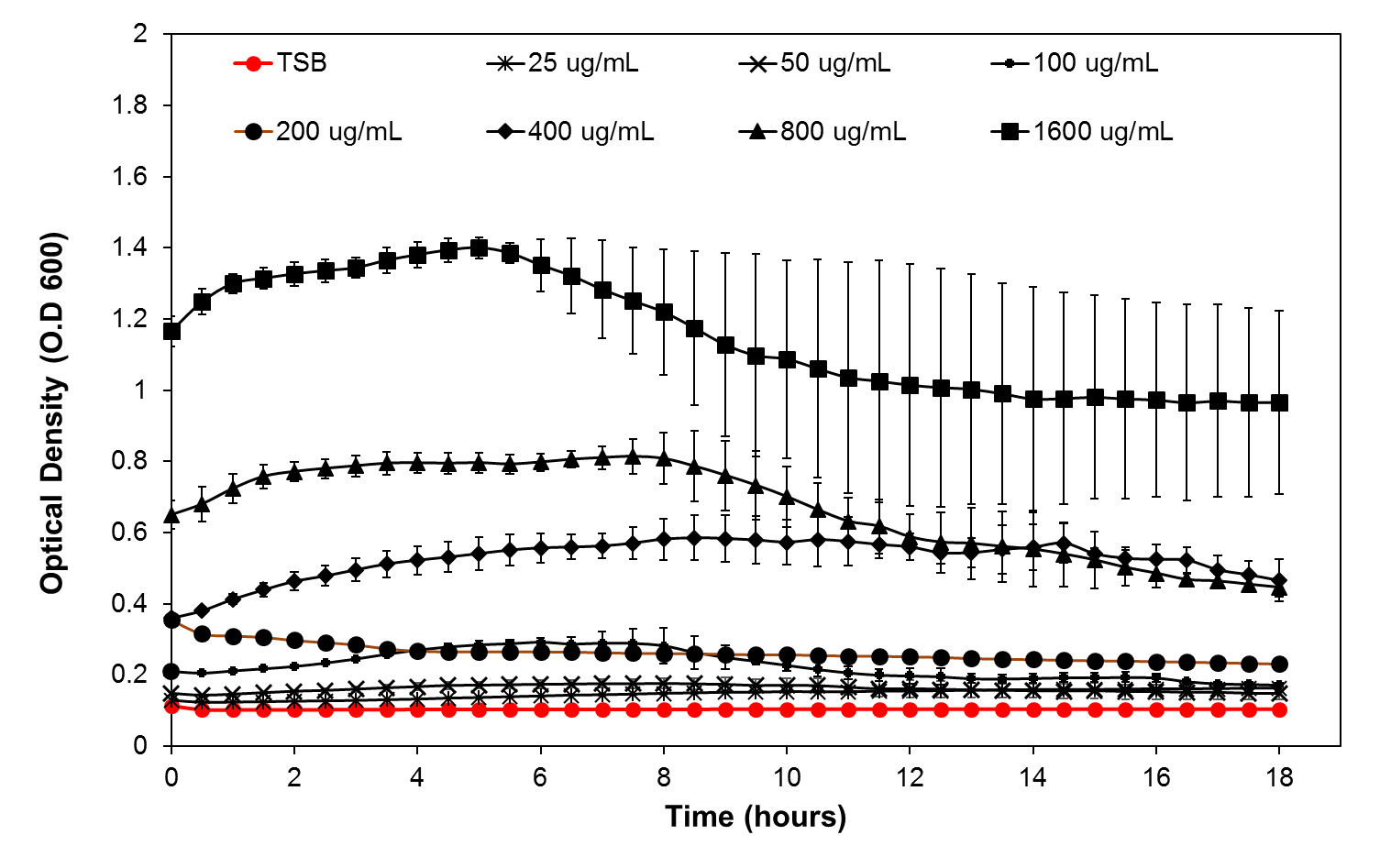


**Figure S1.** The optical density of F-MSN-PEI/Cur. suspensions during 18h. incubation without bacterial cells.

**
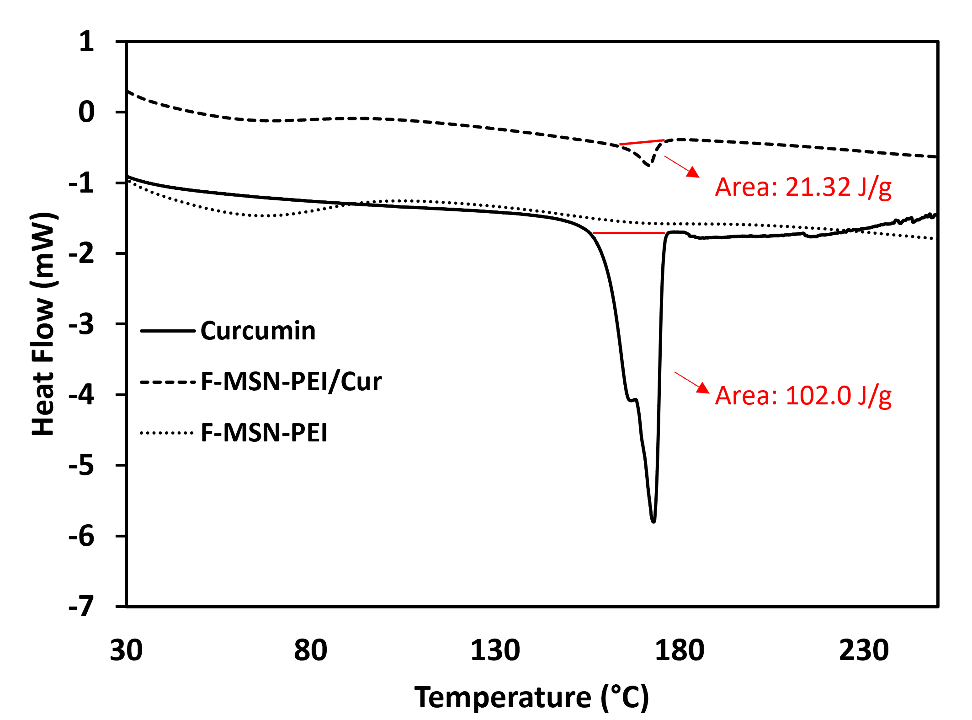
**

**Figure S2**. DSC analysis of curcumin, F-MSN-PEI, F-MSN-PEI/Cur. samples
